# Anti-allergic effects of mapracorat, a novel selective glucocorticoid receptor agonist, in human conjunctival fibroblasts and epithelial cells

**Published:** 2013-07-19

**Authors:** Megan E. Cavet, Stepan Volhejn, Karen L. Harrington, Jin-Zhong Zhang

**Affiliations:** Global Pharmaceutical R&D, Bausch & Lomb Inc., Rochester, New York

## Abstract

**Purpose:**

To determine the ocular anti-allergic effects of mapracorat, a novel selective glucocorticoid receptor agonist (SEGRA) in primary human conjunctival fibroblasts and epithelial cells.

**Methods:**

Two primary human conjunctival cell types, human conjunctival epithelial cells (HConEpiC) and human conjunctival fibroblasts (HConF), were challenged with interleukin-4 (IL-4) or IL-13 plus tumor necrosis factor-alpha (TNF-α). Luminex technology was used to profile the resulting inflammatory response. The effects of mapracorat on the release of eotaxin and regulated on activation, normal T cell expressed and secreted (RANTES), two allergy-related chemokines, as well as proinflammatory cytokines and intercellular adhesion molecule 1 (ICAM-1) were then determined. Small interfering RNA was used to determine whether the effects of mapracorat were mediated via the glucocorticoid receptor (GR). Dexamethasone was used as the control.

**Results:**

IL-13 or IL-4 plus TNF-α in the HConF or HConEpiC significantly increased eotaxin-1 (HConF only), eotaxin-3, RANTES, multiple proinflammatory cytokines, and ICAM-1. Synergistic effects of IL-13 or IL-4 plus TNF-α were observed in the HConEpiC for RANTES and monocyte chemoattractant protein-1, and in the HConF for eotaxin-1, eotaxin-3, and RANTES. Mapracorat significantly reduced IL-4 or IL-13 plus TNF-α-induced cytokine release and ICAM-1 protein in a dose-dependent manner in both cell types, with comparable efficacy to dexamethasone. These effects were mediated through the glucocorticoid receptor (GR), as demonstrated by the reversal of inhibitory effects after silencing of glucocorticoid receptor expression.

**Conclusions:**

Data from these in vitro models indicate that mapracorat is efficacious and potent in reducing IL-4 or IL-13 plus TNF-α-induced release of allergy-related and proinflammatory cytokines from the HConF and the HConEpiC, supporting clinical evaluation of the compound in reducing allergic and inflammatory reactions in allergic conjunctivitis.

## Introduction

Allergic eye disease is a common disorder, affecting 15% to 40% of the population. There are four main types of ocular allergy: seasonal allergic conjunctivitis, perennial allergic conjunctivitis, vernal keratoconjunctivitis (VKC), and atopic keratoconjunctivitis (AKC). While seasonal allergic conjunctivitis and perennial allergic conjunctivitis are self-limiting without threat to vision, VKC and AKC are more severe, chronic diseases with a severe late-phase reaction and may cause damage to sight. Common symptoms of allergic eye disease include itching, tearing, redness, and discomfort, caused by release of histamine, arachidonic acid metabolites, cytokines and chemokines from mast cells, T lymphocytes, and eosinophils, resulting in an inflammatory response within the ocular surface tissues [1-4].

Cytokines and chemokine release from immune cells in the conjunctiva plays a key role in the persistence of inflammation in ocular allergic disease, by eliciting an immune response from the conjunctival epithelium and fibroblasts. In addition to histamine, conjunctival mast cells express and release the proinflammatory cytokines tumor necrosis factor (TNF)-α, interleukin-4 (IL-4), and IL-13 during acute allergic reactions [5-7]. Activated CD4+ T-helper (Th) 2 cells are critical for promoting allergic responses and are increased in the conjunctiva of patients with ocular allergy, releasing cytokines such as IL-4, IL-13, IL-5, and IL-10 [8]. In seasonal allergy, the Th2 cytokines TNF-α and IL-4 are elevated compared to Th1 cytokine levels [9]. In addition, a Th1 response is known to be involved particularly in AKC releasing interferon-γ [10]. Conjunctival epithelial cells and fibroblasts respond to these mediators by upregulating intercellular adhesion molecule 1 (ICAM-1), IL-6, IL-8, eotaxin, regulated and normal T cell expressed and secreted (RANTES), and other cytokines/chemokines [11-16]. This in turn causes further recruitment of more lymphocytes and other activated cell types to the conjunctival epithelia and stroma, exacerbating the inflammatory response [1-4,8].

Therapies for allergic conjunctivitis include mast cell stabilizers, antihistamines, non-steroidal anti-inflammatory drugs, and, for more chronic conditions, corticosteroids [2]. However, long-term ocular use of steroids is limited since they cause side effects such as increased intraocular pressure (IOP) and cataract formation. Selective glucocorticoid receptor agonists (SEGRAs) are a new class of therapeutic designed to have an improved therapeutic index over corticosteroids [17-19]. Mapracorat is a SEGRA that has been demonstrated to selectively reduce inflammation through inhibiting proinflammatory cytokines and activating anti-inflammatory proteins, while having a reduced propensity to cause metabolic and ocular side effects [20-23]. Since conjunctival epithelial cells and fibroblasts are an important source of allergy-related cytokine release, the goals of this study were to establish in vitro conjunctival epithelial and fibroblast ocular allergy models and then determine whether mapracorat is efficacious and potent in reducing allergy-related cytokines in these two ocular cell types.

## Methods

### Reagents

Cell culture media and supplements, Lipofectamine LTX, and Opti-MEM were obtained from Invitrogen (Carlsbad, CA). Fibroblast medium, fibroblast growth supplement (FGS), and poly-L-lysine were purchased from ScienCell (Carlsbad, CA). FNC Coating Mix was from AthenaES (Baltimore, MD). Luminex kits were from Millipore (Billerica, MA). All other reagents were purchased from standard commercial sources and were of the highest available purity.

### Cell cultures

Primary human conjunctival epithelial cells (HConEpiC) and primary human conjunctival fibroblasts (HConF) were received at passage 1 from ScienCell. HConEpiC were maintained in EpiLife medium supplemented with human corneal growth supplement (HCGS, contains bovine pituitary extract, bovine insulin, hydrocortisone, bovine transferrin, and mouse epidermal growth factor), 100 U/ml of penicillin, and 100 µg/ml of streptomycin in FNC Coating Mix coated cultureware at 37 °C in a humidified incubator with 5% CO_2_. Cells were cultured in glucocorticoid-free medium (EpiLife GF free; EpiLife basal medium supplemented with 12.5 µg/ml bovine pituitary extract, 1.25 µg/ml bovine insulin, and 1.25 ng/ml EGF) for 18 h before exposure to IL-4/IL-13 plus TNF-α in basal medium. HConF cells were cultured in complete fibroblast medium, which contained 2% fetal bovine serum and FGS on poly-L-lysine coated cultureware. Cells were used for experiments after reaching confluence (about 95%), and were conditioned in basal Dulbecco's Modified Eagle Medium (DMEM; ATCC, Manassas, VA) for 24 h before treatment.

### Cell treatments

Each treatment was performed in at least triplicate, and appropriate dilutions were prepared to deliver a constant amount of the vehicle to each well. HConEpiC or HConF were seeded on 48-well plates at a density of 2×10^4^ cells per well. To determine the effect of IL-13/IL-4 (1, 10, 100 ng/ml) plus TNF-α (10 ng/ml) on cytokine release and ICAM-1 levels, after the cells became confluent, they were treated with vehicle (0.1% phosphate buffered saline [PBS] plus bovine serum albumin) or IL-4/IL-13 plus TNF-α for 24 h (HConEpiC) or 48 h (HConF). To determine the effect of mapracorat on allergy-related cytokine and ICAM-1 levels, cells were treated with vehicle (0.1% PBS/bovine serum albumin plus 0.1% dimethyl sulfoxide) or IL-4/IL-13 (100 ng/ml) plus TNF-α (10 ng/ml) and 0.1–300 nM mapracorat or dexamethasone.

### Cytokine release and intercellular adhesion molecule 1 expression with multiplex Luminex

Cytokine content in the culture medium and ICAM-1 levels in the cell lysates were analyzed using multiplex Luminex bead technology [24,25] according to the manufacturer's instructions. Cytokine analysis was initially performed on the culture medium measuring eotaxin, granulocyte-colony-stimulating factor, granulocyte-macrophage colony-stimulating factor, IL-1α, IL-1β, IL-2, IL-3, IL-4, IL-5, IL-6, IL-7, IL-8, interferon gamma-inducible protein-10 (IP-10), IP-12p40, IP-12p70, IL-1α, IL-1β, monocyte chemoattractant protein-1 (MCP-1), MCP-3, macrophage inflammatory protein 1 α, macrophage inflammatory protein 1 β, RANTES, TNF-α, cutaneous T cell-attracting chemokine (CTACK), eotaxin 2, eotaxin-3, and IL-11. Once the cytokine release profile after IL-4/IL-13 plus TNF-α for the two cell types was established, only cytokines that demonstrated a significant increase were subsequently assayed for. Briefly, 25 µL of medium or cell lysate samples were incubated with antibody-coated capture beads overnight at 4 °C. Washed beads were further incubated with biotin-labeled antihuman cytokine antibodies for 1 h at room temperature followed by incubation with streptavidin-phycoerythrin for 30 min. Samples were analyzed using Luminex 200 (Luminex, Austin, TX) and Statlia software (Brendan Technologies, Carlsbad, CA). Standard curves of known concentrations of recombinant human cytokines were used to convert median fluorescence intensity to cytokine concentration in pg/mL. Only the linear portions of the standard curves were used to quantify cytokine concentrations.

### Glucocorticoid receptor silencing by small interfering ribonucleic acid

Cells were transfected with 2 pmol negative control or glucocorticoid receptor (GR) small interfering (si)RNA (Santa Cruz Biotechnology, Dallas, TX) using 1 µl Lipofectamine RNAiMax in Opti-MEM media. After 6 h, cells were conditioned in complete medium without glucocorticoids for 18 h. Cells were then treated with basal medium plus 100 ng/ml IL-4 or IL-13 plus 10 ng/ml TNF-α +/− mapracorat or dexamethasone (10 and 30 nM) for 24 h (HConEpiC) or 48 h (HConF). After treatment, culture medium and cell lysates were collected as analyzed with Luminex as described above. Efficient silencing of the GR was confirmed with western blotting using standard techniques. GR was detected using mouse anti-GR monoclonal antibody (Santa Cruz Biotechnology), and equivalent loading was monitored using mouse antiglyceraldehyde-3-phosphate dehydrogenase monoclonal antibody (Invitrogen).

### Statistical analysis

Data were expressed as mean ± standard error of the mean (SEM). Statistical analysis was performed using analysis of variance (ANOVA) with either a Tukey-Kramer or Contrast post-test using the statistical software JMP8 (SAS Institute, Cary, NC). P values less than 0.05 were predetermined to be statistically significant. Data shown are representative of at least two independent experiments. The potential for synergistic effects of combinations of cytokines (IL-4 or IL-13 plus TNF-α) was determined as described [13]. A synergistic effect was defined as an actual effect that was significantly higher than the calculated response.

## Results

### Interleukin-13 and interleukin-4 plus tumor necrosis factor-α induce allergy related cytokines and intercellular adhesion molecule 1 release in conjunctival epithelial cells and fibroblasts

**Figure 1:**
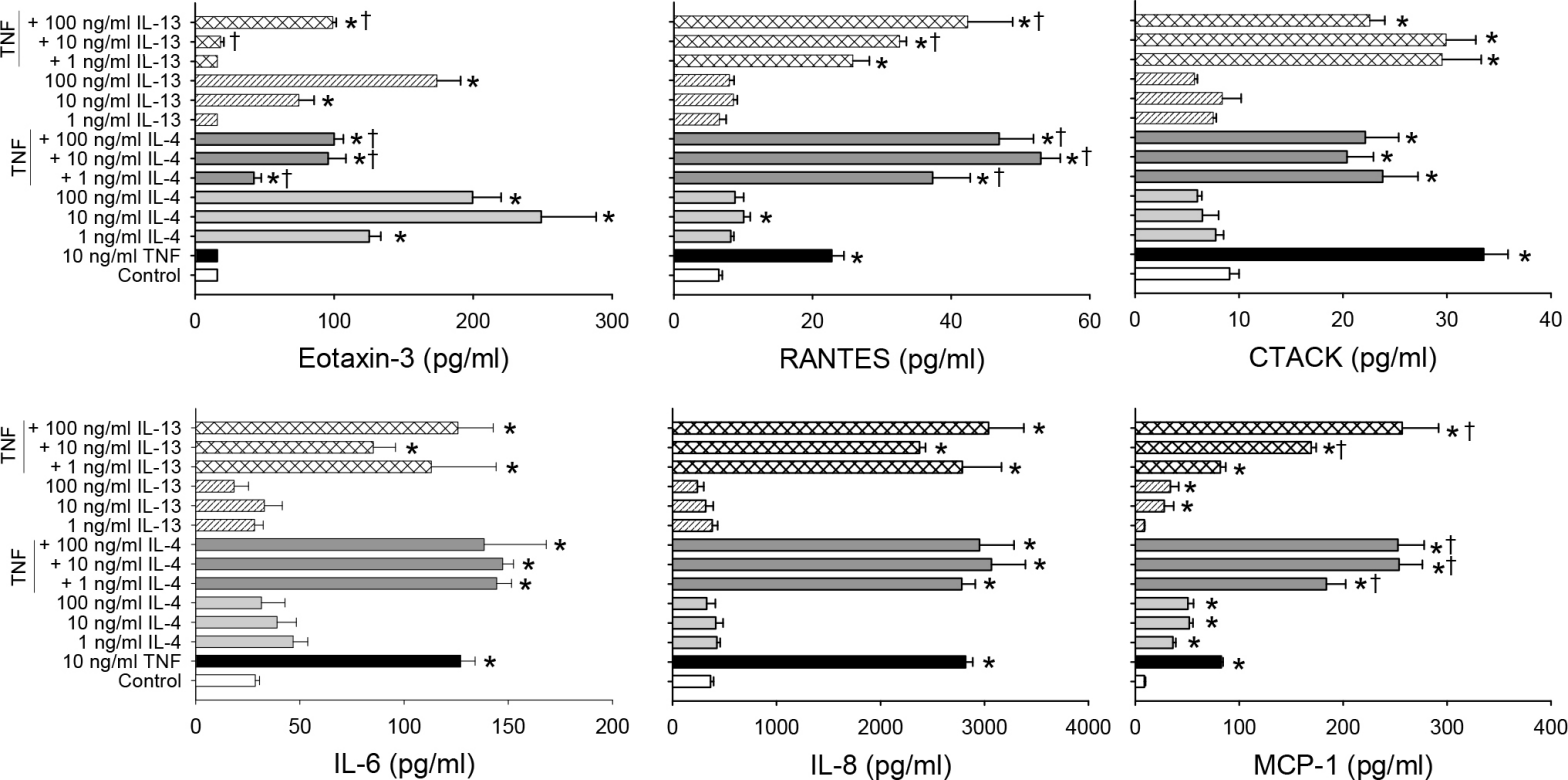
Induction of allergy-related cytokines by interleukin-4/interleukin-13 and tumor necrosis factor-α in human conjunctival epithelial cells. Cells were treated with tumor necrosis factor-α (TNF-α), interleukin-4 (IL-4), and IL-13 alone or in the indicated combinations for 24 h. Cytokine/chemokine levels in the media were analyzed with Luminex. Data are presented as mean ± standard error of the mean (SEM), n=3. * versus control; † *versus* calculated combined effect of IL-4/IL-13 and TNF-α; p<0.05.

To establish in vitro models of allergy-related inflammatory response in conjunctival epithelial cells and fibroblasts, primary cultures of HConEpiC and HConF were exposed to IL-4 or IL-13 alone and in combination with TNF-α. Treatment of HConEpiC with IL-13 or IL-4 alone resulted in an increase in the levels of the allergy-related chemokine eotaxin-3 in the conditioned media at all three doses tested for IL-4 and the highest two doses for IL-13 (10 and 100 ng/ml) after 24 h (Figure 1). TNF-α (10 ng/ml) exposure induced the release of multiple chemokines/cytokines (RANTES, CTACK, IL-6, IL-8, and MCP-1) and an increase in ICAM-1 expression in HConEpiC after 24 h, but did not increase eotaxin-1 levels in the conditioned media (Figure 1 and Table 1). When the HConEpiC were exposed to IL-4 or IL-13 in combination with TNF-α, induction of the majority of cytokines was similar to that observed with TNF-α alone. Two exceptions were RANTES and MCP-1, in which a synergistic increase in release was apparent with IL-4 (at all doses) and IL-13 (at 100 ng/ml), based on a significant increase from the combined calculated values. For eotaxin-3, there was a significant inhibitory effect of a combination of IL-4 (all doses) and IL-13 (10 and 100 ng/ml).

**Table 1:** Induction of cytokines and ICAM-1 by IL-4 or IL-13 + TNF-α in HConEpiC and HConF.

**Inflammatory mediator**	**HConEpiC**					**HConF**				
	**TNF-α**	**IL-4**	**IL-4/ TNF-α**	**IL-13**	**IL-13/ TNF-α**	**TNF-α**	**IL-4**	**IL-4/ TNF-α**	**IL-13**	**IL-13/ TNF-α**
**Eotaxin-1**						+^1^		+ (S)^2^		+ (S)
**Eotaxin-3**		+	+	+	+		+	+ (S)	+	+ (S)
**C-TACK**	+		+		+					
**IL-6**	+		+		+	+	+	+	+	+
**IL-8**	+		+		+	+		+		+
**MCP-1**	+	+	+ (S)	+	+ (S)	+		+		+
**RANTES**	+	+	+ (S)		+ (S)	+		+ (S)		+
**ICAM-1**	+		+	ND^3^	ND	+		+		+ (S)

^1^+ denotes significantly increased over control ^2^S denotes synergistic effect of a combination of IL-4 or IL-13 + TNF-α ^3^ND denotes not determined.

**Figure 2:**
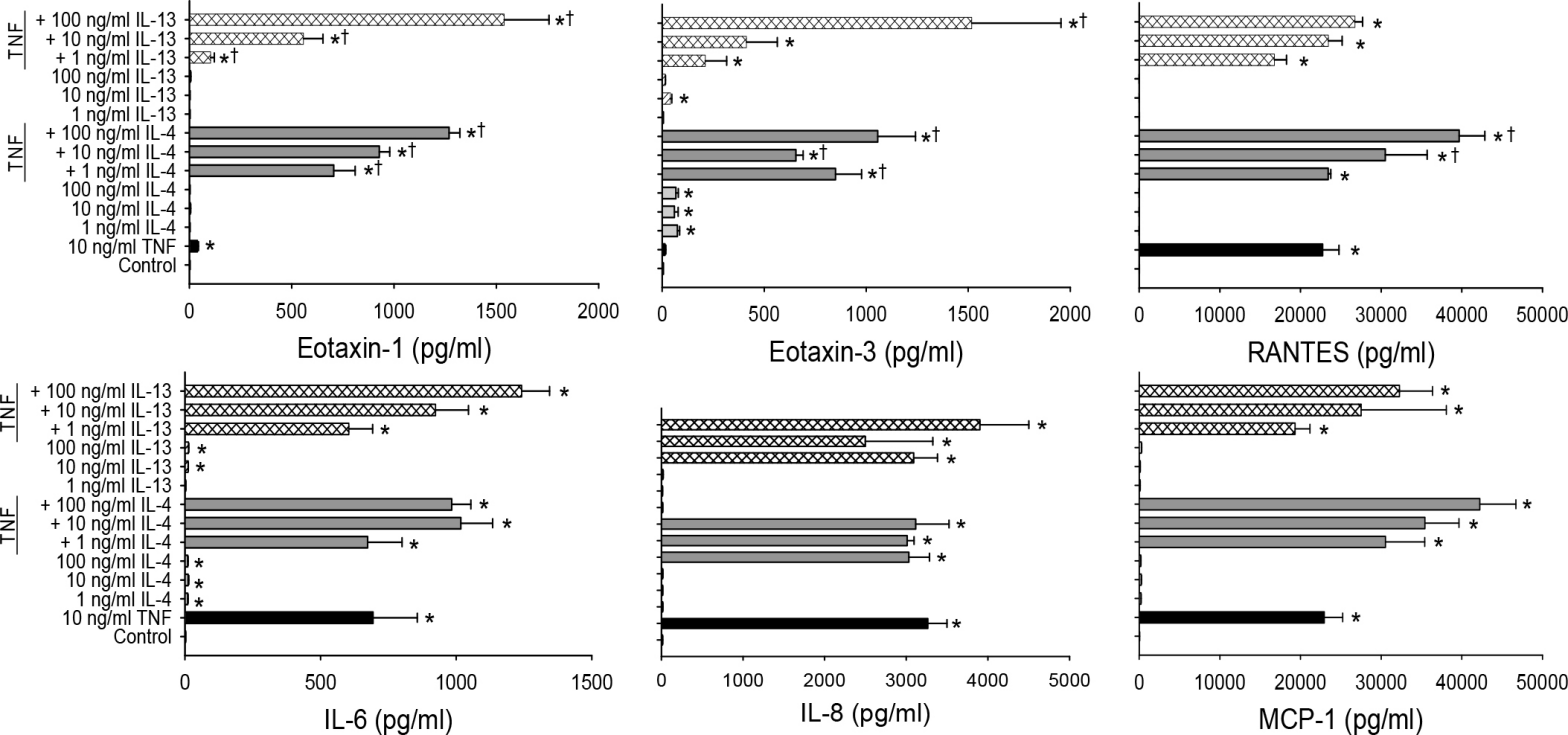
Induction of allergy-related cytokines by interleukin-4/interleukin-13 and tumor necrosis factor-α in human conjunctival fibroblasts. Cells were treated with tumor necrosis factor-α (TNF-α), interleukin-4 (IL-4), and IL-13 alone or in the indicated combinations for 48 h. Cytokine/chemokine levels in the media were analyzed with Luminex. Data are presented as mean±standard error of the mean (SEM), n=3. * versus control; † versus calculated combined effect of IL-4/IL-13 and TNF-α; p<0.05.

When the HConF were exposed to IL-13 or IL-4 alone, there was no effect on eotaxin-1 release, while eotaxin-3 was significantly increased with IL-4 or IL-13. TNF-α produced a robust inflammatory response, increasing eotaxin-1, IL-6, IL-8, ICAM-1, MCP-1, and RANTES release from the HConF (Figure 2 and Table 1). A combination of IL-4 or IL-13 with TNF-α was synergistic in stimulating eotaxin-1 and eotaxin-3, and a synergistic increase in ICAM-1 release was observed with IL-13 and TNF-α in this cell type. In Table 1, the effect of IL-13 or IL-4 alone and in combination with TNF-α in the two cell types is compared. The profile of cytokine/chemokine induction is similar, with a major difference being the lack of eotaxin-1 release by the HConEpiC and the synergistic induction of MCP-1 by IL-13 or IL-4 plus TNF-α in this cell type. In contrast, in the HConF, treatment with IL-13 or IL-4 plus TNF-α resulted in a synergistic increase in eotaxin-1 and -3, RANTES, and ICAM-1; however, no induction of CTACK was observed in this cell type. Based on these results, to determine the effects of mapracorat on allergy-related cytokines, a combination of IL-4 plus TNF-α was selected for the HConEpiC, and IL-13 plus TNF-α was used for the HConF.

### Anti-inflammatory effects of mapracorat in human conjunctival epithelial cells

**Figure 3:**
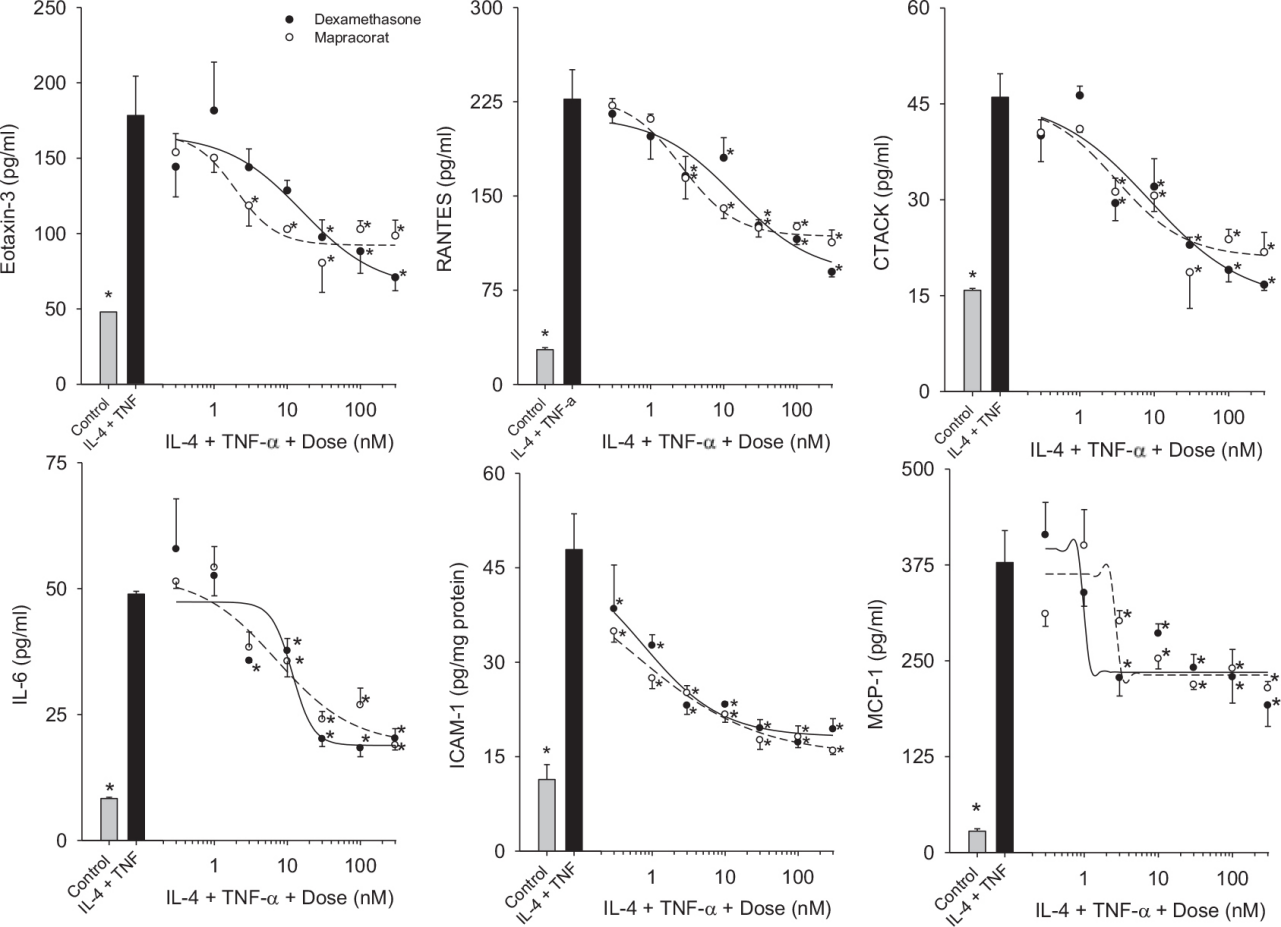
Mapracorat inhibits interleukin-4 plus tumor necrosis factor-α-induced cytokines and intercellular adhesion molecule 1 in human conjunctival epithelial cells. Cells were treated with interleukin-4 (IL-4) plus tumor necrosis factor-α (TNF-α) in the presence of mapracorat or dexamethasone for 24 h. Cytokine/chemokine levels in the media and intercellular adhesion molecule 1 (ICAM-1) in the cell lysates were analyzed with Luminex. Gray bar represents control; black bar represents IL-4 plus TNF-α; black circles plus line represent dexamethasone; white circles plus line represent mapracorat. Lines are the result of a reparameterized four-parameter logistic equation fit to the data. Data are presented as mean ± standard error of the mean (SEM), n=3. * versus IL-4 plus TNF-α; p<0.05.

The anti-inflammatory effects of mapracorat were determined after allergy-related chemokine/cytokine release and ICAM-1 was induced with IL-4 plus TNF-α in HConEpiC. IL-4 plus TNF-α-induced eotaxin-3 levels were significantly decreased by 3, 10, 30, 100, and 300 nM mapracorat and by 30, 100, and 300 nM dexamethasone in a dose-dependent manner (Figure 3). There was a dose-dependent significant reduction in the RANTES, CTACK, and MCP-1 levels measured in the conditioned medium from cells exposed to IL-4 plus TNF-α plus 3–300 nM mapracorat and dexamethasone compared to IL-4 plus TNF-α alone (Figure 3). IL-4 plus TNF-α-induced increases in IL-6 levels in the conditioned medium were significantly reduced with 10–300 nM mapracorat and with 3–300 nM dexamethasone compared to IL-4 plus TNF-α alone (Figure 3). IL-4 plus TNF-α-induced ICAM-1 expression was significantly decreased in the cell lysates after exposure to all doses of mapracorat and dexamethasone (0.3–300 nM) tested (Figure 3). IL-4 plus TNF-α-induced IL-8 levels were significantly decreased comparably by mapracorat and dexamethasone (data not shown).

The half maximal inhibitory concentrations (IC_50s_) for mapracorat and dexamethasone inhibition of IL-4 plus TNF-α-induced cytokine/chemokine and ICAM-1 release/expression (in nM) were as follows: for eotaxin-3, 2.0±1.2 and 16.1±12.9; for IL-6, 7.2±3.9 and 11.8±2.5; for MCP-1, 3.0±0.04 and 1.0±0.1; for RANTES, 2.7±1.0 and 14.2±7.5; for CTACK, 3.1±1.8 and 8.6±7.0, for IL-8, 3.0±1.8 and 11.9±8.5; and for ICAM-1, 0.56±0.27 and 0.75±0.36. Since the 95% confidence limits overlapped, the IC_50s_ were not considered significantly different between mapracorat and dexamethasone. Therefore, these data demonstrate that mapracorat inhibits allergy-related cytokine release with comparable efficacy and potency as dexamethasone in the HConEpiC.

### Mapracorat inhibits interleukin-13 plus tumor necrosis factor-α-induced cytokine release in human conjunctival fibroblasts

**Figure 4:**
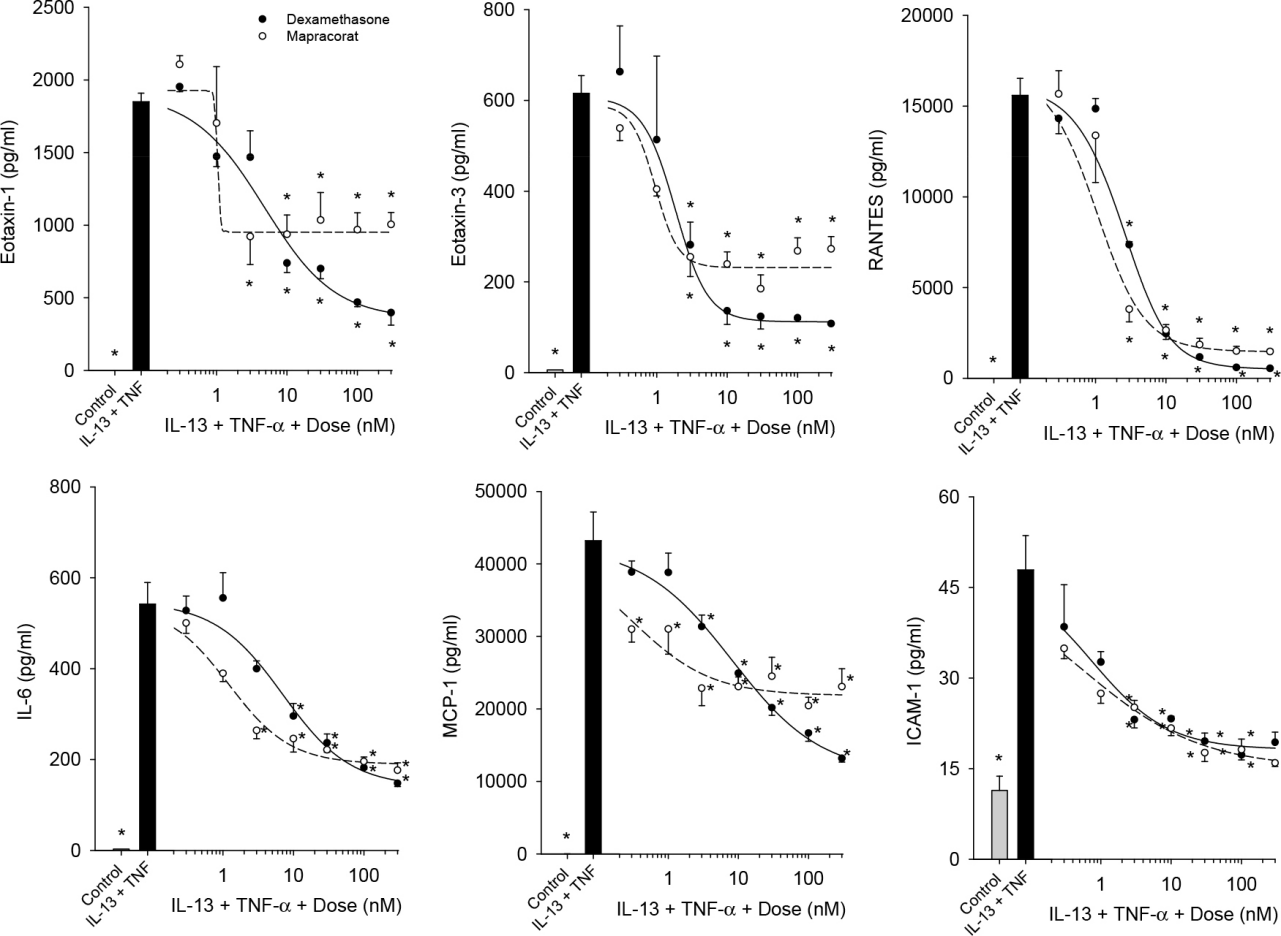
Mapracorat inhibits interleukin-13 plus tumor necrosis factor-α-induced cytokines and intercellular adhesion molecule 1 in human conjunctival fibroblasts. Cells were treated with interleukin-13 (IL-13) plus tumor necrosis factor-α (TNF-α) in the presence of mapracorat or dexamethasone for 48 h. Cytokine and intercellular adhesion molecule 1 (ICAM-1) levels in the media were analyzed with Luminex. Gray bar represents control; black bar represents IL-13 plus TNF-α; black circles plus line represent dexamethasone; white circles plus line represent mapracorat. Lines are the result of a reparameterized four-parameter logistic equation fit to the data. Data are presented as mean ± standard error of the mean (SEM), n=3. * versus IL-13 plus TNF-α; p<0.05.

The anti-inflammatory effects of mapracorat on eotaxin-1, eotaxin-3, RANTES, IL-6, IL-8, MCP-1, and ICAM-1 were investigated in the HConF after exposure to 10 ng/ml TNF-α and 100 ng/ml IL-13. Significant inhibition of IL-13 plus TNF-α induced eotaxin-1 and IL-6 release was observed with doses of 3–300 nM for mapracorat and 10–300 nM for dexamethasone (Figure 4). Significant dose-dependent decreases of induced eotaxin-3, RANTES, and ICAM-1 levels were observed with 3–300 nM doses of both drugs (Figure 4). Significant dose-dependent decreases of induced MCP-1 release were observed with all doses of mapracorat tested and 3, 10, 30, 100, and 300 nM doses of dexamethasone (Figure 4). IL-8 was similarly inhibited by both drugs (data not shown). Maximal efficacy of mapracorat was comparable to dexamethasone for the majority of cytokines, though slightly lower for mapracorat compared to dexamethasone in the case of eotaxin-1.

As was found for the HConEpiC, the IC_50s_ for mapracorat and dexamethasone inhibition of IL-13 plus TNF-α-induced cytokine/chemokine and ICAM-1 release/expression in the HConF were not considered significantly different between mapracorat and dexamethasone, since the 95% confidence limits did not overlap, except with MCP-1 where the IC_50_ for mapracorat was lower than for dexamethasone. The IC_50s_ (in nM) were as follows: for eotaxin-1, 1.0±0.1 and 4.7±1.6; for eotaxin-3, 0.9±0.3 and 1.8±0.7; for RANTES, 1.0±0.5 and 2.4±0.6; for IL-6, 1.2±0.4 and 7.0±2.2; for MCP-1, 0.3±0.3 and 8.0±3.3; for IL-8, 2.4±7.7 and 10.7±4.4; and for ICAM-1 7.2±3.9 and 13.4±4.7 for mapracorat and dexamethasone respectively.

### Mapracorat suppresses allergy-related cytokine release and intercellular adhesion molecule 1 expression via the glucocorticoid receptor in human conjunctival epithelial cells and human conjunctival fibroblasts

**Figure 5:**
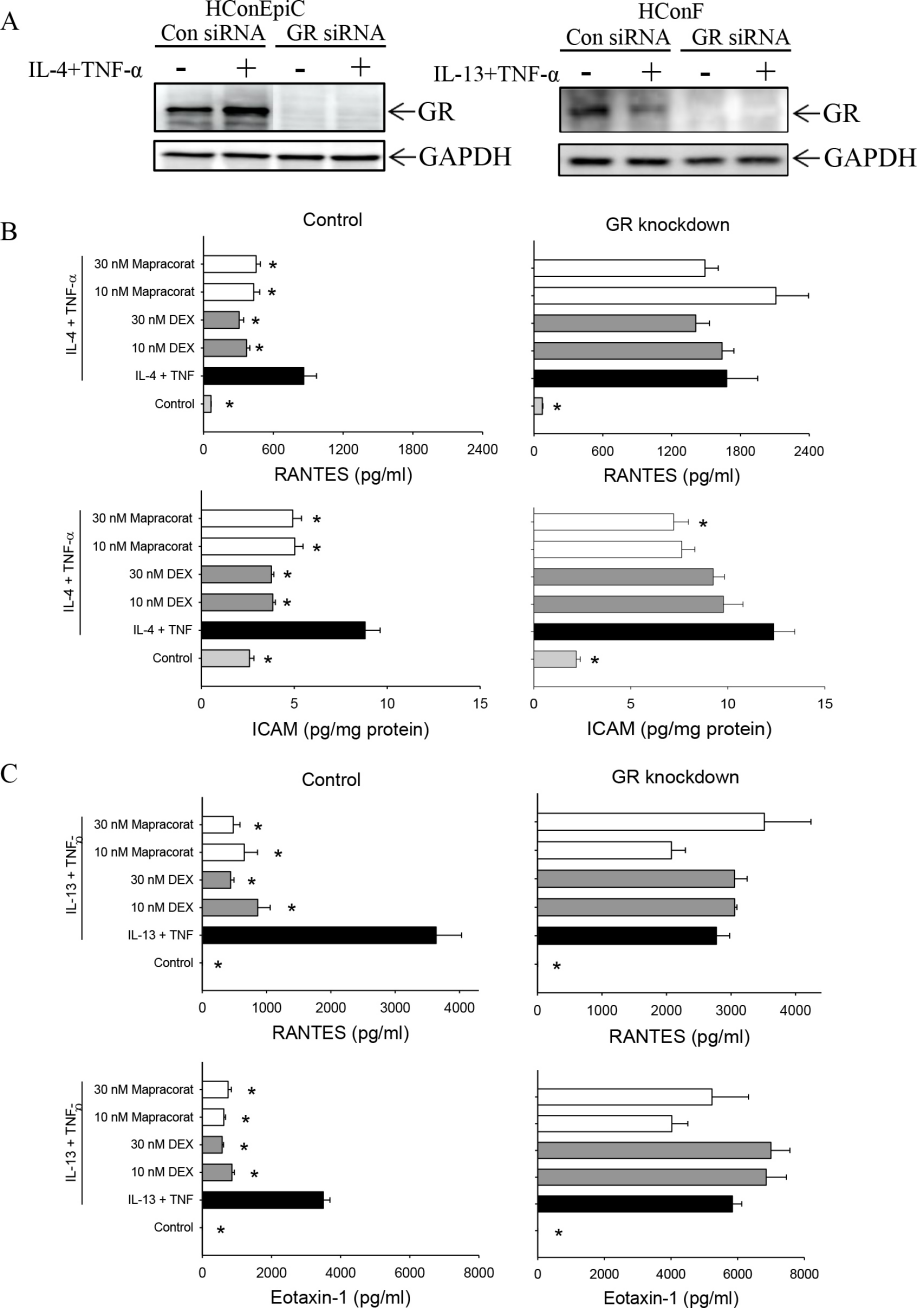
Effect of glucocorticoid receptor silencing on mapracorat inhibition of interleukin-4/interleukin-13 plus tumor necrosis factor-α-induced cytokines and intercellular adhesion molecule 1 in human conjunctival epithelial cells and human conjunctival fibroblasts. After glucocorticoid receptor (GR) knockdown, cells were treated with interleukin-4 (IL-4) plus tumor necrosis factor-α (TNF-α) (human conjunctival epithelial cells [HConEpiC]) or IL-13 plus TNF-α (human conjunctival fibroblasts [HConF]) in the presence of mapracorat or dexamethasone. **A**: GR protein expression was determined with western blotting. **B**: Regulated on activation, normal T cell expressed and secreted (RANTES) levels and intercellular adhesion molecule 1 (ICAM-1) expression in HConEpiC were determined by Luminex. **C**: RANTES and eotaxin-1 levels in HConF were determined by Luminex. Left panels show data for control small interfering RNA (siRNA); right panels show data for GR siRNA. Data are presented as mean ± standard error of the mean (SEM), n=4. * versus IL-4/IL-13 plus TNF-α; p<0.05.

To determine whether the anti-allergic effects of mapracorat were mediated by the GR, the effect of GR silencing on allergy-induced cytokine release and ICAM-1 expression was determined. Efficient silencing of the GR was confirmed with western blotting in both cell types (Figure 5A). GR siRNA knockdown was similar in both cell types: 89.5±1.6% in the HConEpiC and 89.9±3.0% in the HConF. Figure 5B shows that there was a significant decrease in the IL-4 plus TNF-α induced levels of RANTES and ICAM-1 with 10 and 30 nM dexamethasone or mapracorat compared to IL-4 plus TNF-α alone in the control siRNA transfected HConEpiC. In contrast, in the GR siRNA transfected HConEpiC, neither dexamethasone nor mapracorat had a significant effect on IL-4 plus TNF-α induced RANTES (Figure 5B). Similarly, there was a significant decrease in IL-4 plus TNF-α-induced ICAM-1 only with 30 nM mapracorat in the GR knockdown HConEpiC (Figure 5B). Similar results were obtained for other induced cytokines (IL-6, IL-8, and MCP-1; data not shown). Comparable results were obtained in the HConF. Figure 5C shows that the inhibitory effects of mapracorat and dexamethasone on RANTES and eotaxin-1 were abrogated when the GR was knocked down by siRNA. The effects of the GR siRNA knockdown were similar for other measured cytokines (eotaxin-3 and IL-6). These data indicate that the anti-allergic effects of mapracorat in HConEpiC and HConF are mediated through the GR.

## Discussion

The major finding of this study is that the novel SEGRA mapracorat inhibits allergy-related chemokine/cytokine release and the expression of ICAM-1 in two ocular cell types, HConEpiC and HConF. The potency and efficacy of the inhibitory effects were similar to those of the highly effective anti-inflammatory steroid dexamethasone. Data obtained in this study support findings from a recent in vivo study, which determined that topical mapracorat reduced the composite score for clinical signs of conjunctivitis (tearing, redness, and edema) as well as the infiltration of eosinophils into the conjunctiva of guinea pigs with ocular allergy [26]. That study also showed that mapracorat inhibited eosinophil migration and proinflammatory release from eosinophils and mast cells. The current study indicates that the observed in vivo anti-allergic effects are mediated in part via an anti-inflammatory effect of mapracorat directly on conjunctival epithelial cells and fibroblasts. In the present study, key cytokines involved in allergic conjunctivitis were used to upregulate inflammatory mediators. IL-4/IL-13 are present in conjunctival mast cells, in addition to T lymphocytes, while the proinflammatory cytokine TNF-α is also released from conjunctival mast cells in an allergic response [5-8]. Further studies could address the response of cells to other known allergy-related cytokines such as interferon-γ.

SEGRAs, like glucocorticoids, are designed to transrepress genes primarily through tethering type mechanisms, and thus have potent anti-inflammatory activity. However, in contrast to glucocorticoids, SEGRAs are selected to have a decreased ability to activate gene transcription through binding to simple glucocorticoid responsive elements, and thus have a lower ability to induce unwanted side effects [17-19]. Mapracorat has previously been shown to mediate potent anti-inflammatory effects in multiple ocular cell types. Mapracorat inhibits proinflammatory cytokine and chemokine production in human corneal epithelial cells, human corneal fibroblasts, and the HConF after IL-1β challenge [27]. In an in vitro HConEpiC osmotic stress model, which mimics some of the pathophysiological changes seen in dry eye syndrome, mapracorat inhibited hyperosmolar-induced cytokine release [28]. In an atropine-induced dry eye model in rabbits, topical mapracorat inhibited the atropine-induced effects on tear production and tear film break-up time with comparable efficacy as dexamethasone [23]. In the paracentesis model of anterior segment inflammation in rabbits, topical mapracorat inhibited the formation of prostaglandin E_2_ and accumulation of protein and leukocytes in the aqueous humor and myeloperoxidase activity in the iris/ciliary body. No large difference in activity was observed between mapracorat and the positive controls (dexamethasone, loteprednol etabonate, or flurbiprofen) [23]. In addition to the anti-inflammatory efficacy in ocular models, the in vivo anti-inflammatory activity of mapracorat was demonstrated in two different models of skin inflammation (irritant contact dermatitis and allergic contact dermatitis) in mice and rats [29].

Glucocorticoid receptor silencing studies using siRNA demonstrated that the antiallergic effects of mapracorat in HConEpiC and HConF are mediated predominantly via the GR. Inhibition of nuclear factor kappa B and activator protein-1 transcriptional activation and upstream mitogen-activated protein kinase signaling likely contribute to the inhibitory effects of mapracorat on cytokine release and ICAM-1 expression in the two cell types. These mechanisms are well-known as contributing to the anti-inflammatory effects of glucocorticoids [19,30] and have been demonstrated to occur with mapracorat after hyperosmolar or IL-1β challenge in corneal epithelial cells [27,28]. Mapracorat probably also targets other transcription factors to mediate its anti-allergic effects. Signal transducer and activator of transcription 6, interleukin-4 is known to be involved in the upregulation of eotaxin in response to IL-4 or IL-13 [31], and the SEGRA Compound A has been shown to inhibit the translocation of this transcription factor to the nucleus, in a similar manner to dexamethasone in a lung model of acute inflammation [32]. The exact mechanisms by which mapracorat inhibits allergy-related cytokines in the HConEpiC and HConF is an area for future studies.

Previous in vitro and in vivo studies have determined that mapracorat has a reduced side effect profile compared to steroids, both systemically and in the ocular setting [22,23,26,29]. Particularly important for ocular use, mapracorat demonstrated a decreased activation profile for the glaucoma-related protein myocilin compared with dexamethasone in trabecular meshwork cells [22]. In addition, IOP increases were not evident in a rabbit model with dosing four times a day over a period of 6 weeks, while dexamethasone significantly elevated IOP [23]. These data indicate that mapracorat may have less potential for inducing glaucoma than traditional glucocorticoids in vivo. In addition, ocular topical application of mapracorat to the ocular surface did not result in a significant weight loss, in contrast to the steroid dexamethasone in a rabbit model [23]. Mapracorat demonstrated less activation than classical steroids in activating the prodiabetogenic enzyme tyrosine aminotransferase when administered subcutaneously in rats. In addition, topical administration did not result in disturbed tolerance to oral glucose challenge, in contrast to the elevated glucose levels observed with classical steroids [29]. Collectively, data demonstrating anti-allergic effects and a reduced induction of side effects support clinical evaluation of mapracorat in reducing inflammation in allergic eye disease.

An additional component of this study was a direct comparison of the cytokines induced by the allergy-related mediators IL-4/IL-13 with TNF-α in two surface ocular cell types, HConEpiC and HConF. We observed an increase in the allergy-related cytokines RANTES and eotaxin-3 in the HConEpiC and the HConF, but in contrast, eotaxin-1 was increased only in the HConF, not in the HConEpiC. A novel finding was the induction of eotaxin-3 by IL-4 and IL-13 in the HConEpiC and the HConF. This cytokine has been reported to be involved in the allergic response in VKC and is an eosinophil attractant [33]. Eotaxin-1 has been found in the tears and mucus of allergy patients with corneal ulcer and in the tears of patients with seasonal allergy, and, like eotaxin-3, is chemotactic for eosinophils [12,34,35]. RANTES is also increased in the tears and conjunctival tissues of allergy patients [11,14]. An additive effect of a combination of IL-4/IL-13 plus TNF-α was apparent for the majority of cytokines in the two cell types, the HConEpiC and the HConF, tested in this study. Except eotaxin-3, IL-4 and IL-13 alone did not elicit a large cytokine response in the HConEpiC but a combination of IL-4 plus TNF-α resulted in synergistic effect on RANTES and MCP-1 release in this cell type. In the HConF, a synergistic effect of IL-13 and TNF-α was observed for eotaxin-1 and eotaxin-3 and ICAM-1. In previously published studies, synergistic effects were obtained with a combination of TNF-α and IL-4 in stimulating eotaxin-1 and VCAM-1 levels in corneal fibroblasts [36,37].

In summary, these data demonstrate that mapracorat acts as a potent anti-inflammatory agent in HConEpiC and HConF challenged with allergy-related cytokines. Previous studies have shown that mapracorat has a reduced propensity to cause metabolic and ocular side effects compared to steroids such as dexamethasone and prednisolone acetate. Therefore, mapracorat has potential therapeutic application in treating allergic eye disease.

## References

[1] Ono SJ, Abelson MB (2005). Allergic conjunctivitis: update on pathophysiology and prospects for future treatment.. J Allergy Clin Immunol.

[2] Bielory BP, Perez VL, Bielory L (2010). Treatment of seasonal allergic conjunctivitis with ophthalmic corticosteroids: in search of the perfect ocular corticosteroids in the treatment of allergic conjunctivitis.. Curr Opin Allergy Clin Immunol.

[3] Offiah I, Calder VL (2009). Immune mechanisms in allergic eye diseases: what is new?. Curr Opin Allergy Clin Immunol.

[4] Bielory L (2008). Ocular allergy overview.. Immunol Allergy Clin North Am.

[5] Macleod JD, Anderson DF, Baddeley SM, Holgate ST, McGill JI, Roche WR (1997). Immunolocalization of cytokines to mast cells in normal and allergic conjunctiva.. Clin Exp Allergy.

[6] Anderson DF, Zhang S, Bradding P, McGill JI, Holgate ST, Roche WR (2001). The relative contribution of mast cell subsets to conjunctival TH2-like cytokines.. Invest Ophthalmol Vis Sci.

[7] Cook EB, Stahl JL, Barney NP, Graziano FM (2001). Olopatadine inhibits anti-immunoglobulin E-stimulated conjunctival mast cell upregulation of ICAM-1 expression on conjunctival epithelial cells.. Ann Allergy Asthma Immunol.

[8] Kumagai N, Fukuda K, Fujitsu Y, Yamamoto K, Nishida T (2006). Role of structural cells of the cornea and conjunctiva in the pathogenesis of vernal keratoconjunctivitis.. Prog Retin Eye Res.

[9] Cook EB, Stahl JL, Lowe L, Chen R, Morgan E, Wilson J, Varro R, Chan A, Graziano FM, Barney NP (2001). Simultaneous measurement of six cytokines in a single sample of human tears using microparticle-based flow cytometry: allergics vs. non-allergics.. J Immunol Methods.

[10] Stern ME, Siemasko K, Gao J, Duong A, Beauregard C, Calder V, Niederkorn JY (2005). Role of interferon-gamma in a mouse model of allergic conjunctivitis.. Invest Ophthalmol Vis Sci.

[11] Leonardi A, Curnow SJ, Zhan H, Calder VL (2006). Multiple cytokines in human tear specimens in seasonal and chronic allergic eye disease and in conjunctival fibroblast cultures.. Clin Exp Allergy.

[12] Leonardi A, Jose PJ, Zhan H, Calder VL (2003). Tear and mucus eotaxin-1 and eotaxin-2 in allergic keratoconjunctivitis.. Ophthalmology.

[13] Stahl JL, Cook EB, Graziano FM, Barney NP (2003). Differential and cooperative effects of TNFalpha, IL-1beta, and IFNgamma on human conjunctival epithelial cell receptor expression and chemokine release.. Invest Ophthalmol Vis Sci.

[14] Hingorani M, Calder VL, Buckley RJ, Lightman SL (1998). The role of conjunctival epithelial cells in chronic ocular allergic disease.. Exp Eye Res.

[15] Fukagawa K, Okada N, Fujishima H, Nakajima T, Takano Y, Tanaka M, Dogru M, Satake Y, Tsubota K, Saito H (2009). Corneal and conjunctival fibroblasts are major sources of eosinophil-recruiting chemokines.. Allergol Int.

[16] Enríquez-de-Salamanca A, Calder V, Gao J, Galatowicz G, Garcia-Vazquez C, Fernandez I, Stern ME, Diebold Y, Calonge M (2008). Cytokine responses by conjunctival epithelial cells: an in vitro model of ocular inflammation.. Cytokine.

[17] De Bosscher K (2010). Selective Glucocorticoid Receptor modulators.. J Steroid Biochem Mol Biol.

[18] Stahn C, Lowenberg M, Hommes DW, Buttgereit F (2007). Molecular mechanisms of glucocorticoid action and selective glucocorticoid receptor agonists.. Mol Cell Endocrinol.

[19] Löwenberg M, Stahn C, Hommes DW, Buttgereit F (2008). Novel insights into mechanisms of glucocorticoid action and the development of new glucocorticoid receptor ligands.. Steroids.

[20] Schäcke H, Berger M, Rehwinkel H, Asadullah K (2007). Selective glucocorticoid receptor agonists (SEGRAs): novel ligands with an improved therapeutic index.. Mol Cell Endocrinol.

[21] Schäcke H, Hennekes H, Schottelius A, Jaroch S, Lehmann M, Schmees N, Rehwinkel H, Asadullah K (2002). SEGRAs: a novel class of anti-inflammatory compounds.. Ernst Schering Res Found Workshop.

[22] Pfeffer BA, DeWitt CA, Salvador-Silva M, Cavet ME, Lopez FJ, Ward KW (2010). Reduced myocilin expression in cultured monkey trabecular meshwork cells induced by a selective glucocorticoid receptor agonist: comparison with steroids.. Invest Ophthalmol Vis Sci.

[23] Shafiee A, Bucolo C, Budzynski E, Ward KW, Lopez FJ (2011). In vivo ocular efficacy profile of mapracorat, a novel selective glucocorticoid receptor agonist, in rabbit models of ocular disease.. Invest Ophthalmol Vis Sci.

[24] Vignali DA (2000). Multiplexed particle-based flow cytometric assays.. J Immunol Methods.

[25] Morgan E, Varro R, Sepulveda H, Ember JA, Apgar J, Wilson J, Lowe L, Chen R, Shivraj L, Agadir A, Campos R, Ernst D, Gaur A (2004). Cytometric bead array: a multiplexed assay platform with applications in various areas of biology.. Clin Immunol.

[26] Baiula M, Sparta A, Bedini A, Carbonari G, Bucolo C, Ward KW, Zhang JZ, Govoni P, Spampinato S (2011). Eosinophil as a cellular target of the ocular anti-allergic action of mapracorat, a novel selective glucocorticoid receptor agonist.. Mol Vis.

[27] Zhang JZ, Cavet ME, VanderMeid KR, Salvador-Silva M, Lopez FJ, Ward KW (2009). BOL-303242-X, a novel selective glucocorticoid receptor agonist, with full anti-inflammatory properties in human ocular cells.. Mol Vis.

[28] Cavet ME, Harrington KL, Ward KW, Zhang JZ (2010). Mapracorat, a novel selective glucocorticoid receptor agonist, inhibits hyperosmolar-induced cytokine release and MAPK pathways in human corneal epithelial cells.. Mol Vis.

[29] Schäcke H, Zollner TM, Docke WD, Rehwinkel H, Jaroch S, Skuballa W, Neuhaus R, May E, Zugel U, Asadullah K (2009). Characterization of ZK 245186, a novel, selective glucocorticoid receptor agonist for the topical treatment of inflammatory skin diseases.. Br J Pharmacol.

[30] Beck IM, Vanden Berghe W, Vermeulen L, Yamamoto KR, Haegeman G, De Bosscher K (2009). Crosstalk in inflammation: the interplay of glucocorticoid receptor-based mechanisms and kinases and phosphatases.. Endocr Rev.

[31] Hebenstreit D, Wirnsberger G, Horejs-Hoeck J, Duschl A (2006). Signaling mechanisms, interaction partners, and target genes of STAT6.. Cytokine Growth Factor Rev.

[32] Reber LL, Daubeuf F, Plantinga M, De Cauwer L, Gerlo S, Waelput W, Van Calenbergh S, Tavernier J, Haegeman G, Lambrecht BN, Frossard N, De Bosscher K (2012). A dissociated glucocorticoid receptor modulator reduces airway hyperresponsiveness and inflammation in a mouse model of asthma.. J Immunol.

[33] Shoji J, Inada N, Sawa M (2009). Evaluation of eotaxin-1, −2, and −3 protein production and messenger RNA expression in patients with vernal keratoconjunctivitis.. Jpn J Ophthalmol.

[34] Fukagawa K, Nakajima T, Tsubota K, Shimmura S, Saito H, Hirai K (1999). Presence of eotaxin in tears of patients with atopic keratoconjunctivitis with severe corneal damage.. J Allergy Clin Immunol.

[35] Eperon S, Sauty A, Lanz R, Leimgruber A, Lurati F, Guex-Crosier Y (2004). Eotaxin-1 (CCL11) up-regulation in tears during seasonal allergic conjunctivitis.. Graefes Arch Clin Exp Ophthalmol.

[36] Kumagai N, Fukuda K, Ishimura Y, Nishida T (2000). Synergistic induction of eotaxin expression in human keratocytes by TNF-alpha and IL-4 or IL-13.. Invest Ophthalmol Vis Sci.

[37] Kumagai N, Fukuda K, Fujitsu Y, Nishida T (2003). Synergistic effect of TNF-alpha and either IL-4 or IL-13 on VCAM-1 expression by cultured human corneal fibroblasts.. Cornea.

